# Proton Conduction via Water Bridges Hydrated in the Collagen Film

**DOI:** 10.3390/jfb11030061

**Published:** 2020-09-02

**Authors:** Hiroshi Matsui, Yasumitsu Matsuo

**Affiliations:** 1 Department of Physics, Graduate School of Science, Tohoku University, Sendai 980-8578, Japan; 2 Department of Life Science, Faculty of Science & Engineering, Setsunan University, Neyagawa 572-8508, Japan; ymatsuo@lif.setsunan.ac.jp

**Keywords:** proton conduction, ionic conduction, fuel-cell electrolyte, collagen, hydration, water bridge, hydrogen bond, biomaterial, biofuel cell, bio-electrolyte

## Abstract

Collagen films with proton conduction are a candidate of next generation of fuel-cell electrolyte. To clarify a relation between proton conductivity and formation of water networks in the collagen film originating from a tilapia’s scale, we systematically measured the ac conductivity, infrared absorption spectrum, and weight change as a function of relative humidity (RH) at room temperature. The integrated absorbance concerning an O–H stretching mode of water molecules increases above 60% RH in accordance with the weight change. The dc conductivity varies in the vicinity of 60 and 83% RH. From those results, we have determined the dc conductivity vs. hydration number (*N*) per unit (Gly-X-Y). The proton conduction is negligible in the collagen molecule itself, but dominated by the hydration shell, the development of which is characterized with three regions. For 0 < *N* < 2, the conductivity is extremely small, because the water molecule in the primary hydration shell has a little hydrogen bonded with each other. For 2 < *N* < 4, a quasi-one-dimensional proton conduction occurs through intra-water bridges in the helix. For 4 < *N*, the water molecule fills the helix, and inter-water bridges are formed in between the adjacent helices, so that a proton-conducting network is extended three dimensional.

## 1. Introduction

Proton conduction is generated not only in many solid electrolytes [1], but also in various biological materials such as proteins (collagen), polysaccharides (chitin and chitosan), and DNAs [2,3,4,5,6,7,8]. Many attempts have been done to apply biological materials to novel bioprotonic devices such as a FET, humid sensor, and artificial neuron [9,10,11]. Among them, a collagen contained in skins, bones, and scales is desired to be reused in light of effective utilization of food wastes. Collagen films with sufficient toughness are found to have a functionality of fuel-cell electrolyte as well as in Nafion [3]. Once the Nafion film is dehydrated, the performance is hugely reduced owing to a structural damage. In contrast, collagen films have no damage even in evacuation, and the functionality is recovered by rehydration. Due to the high stability, treatment of the film becomes easy for various operations. Nevertheless, it is not clear how the hydration in collagen films is formed and contributes to the proton conduction.

Proton conducting phenomena in perovskite oxides [12,13] and *M*_3_H(SeO_4_)_2_ (*M* = K, Rb, Cs) [14] have been extensively studied so far to explore solid fuel-cell electrolytes without water molecules. The conduction is investigated in terms of a protonic polaron [15] or phonon-assisted proton tunneling [14], which originate from a proton-lattice (phonon) interaction [16]. Various protonic states in those materials are worth studying from a physics point of view, though the practicability is not so high at present, because the conductivity is too low at room temperature in contrast to Nafion and the corresponding polymers involving plenty of water molecules.

**Figure 1:**
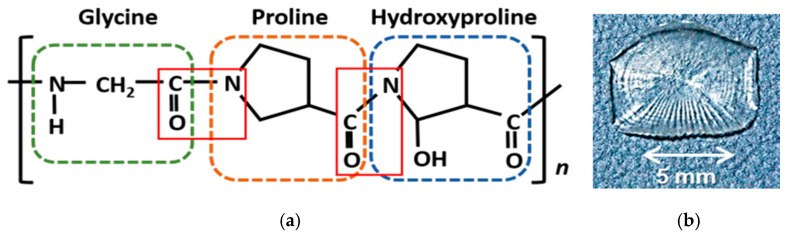
α helix consisting of Gly-Pro-Hyp unit connected by a peptide linkage marked with red squares (**a**); picture of the collagen film obtained from a tilapia’s scale (**b**).

A proton transfer via water networks has been widely studied, for instance, in water nanotubes and water chains confined in the hydrophilic nanochannel of molecular porous crystals [17,18,19]. The proton is affected by the size, geometry, and interfacial interaction arising from the framework molecules. Furthermore, Xe hydrates in the water nanotube inhibit the proton conduction owing to the hardening and reconstruction of hydrogen-bonding networks [20]. The porous environment has a significant influence on the conducting properties. As for collagen, the proton conduction may be strongly affected by the hydration state that is formed in and out of the triple helix [21] regarded as a framework molecule. A single α helix has a peptide-bonding unit basically consisting of glycine (Gly) and another two amino acids. Figure 1a illustrates the unit composed of glycine, proline (Pro), and hydroxyproline (Hyp).

A single crystal of collagen is difficult to synthesize, whereas the X-ray structural analysis has been performed with the crystal of collagen-like peptides such as (Gly-Hyp-Pro)_5_-(Ala-Hyp-Pro)(Gly-Hyp-Pro)_4_ [22], where Ala denotes an alanine. In the primary hydration shell, water molecules are hydrogen bonded to N–H of Gly, O–H of Hyp, and C = O in the peptide linkage. Intra-water bridges (1–4 water molecules) in between those groups are identified inside of the triple helix [23,24,25,26]. Moreover, the helix is connected to the adjacent helices through an inter-water bridge. A collagen film is expected to have identical hydration structures and water bridges to the peptide crystal. Instead of X-ray diffraction experiments, we are available to examine the hydration state employing an infrared absorbance spectrum [27]. Together with the measurements on conductivity and weight change, we have demonstrated that the development of intra- and inter-water bridges in the collagen film enhances the proton conduction.

## 2. Samples and Experimental Methods

Filmy collagen samples that originated from a tilapia’s scale were purchased from Nitta Gelatin Inc. (Osaka, Japan). The sample was obtained by a decalcification together with conventional purification processes of pickling and neutralization [3]. As shown in Figure 1b, the translucent sample remains a scale’s pattern. We are available to remove almost all the impurities such as other proteins and metallic elements. As mentioned later, the present sample has the typical proton conductivity of 10^−3^ S/m (90% RH) that is comparable to one for collagen films that originated from a sea-bream scale and submucosa [28]. The coincidence suggests that the sample preparation has a little effect on the result of proton conductivity and hydration state. In addition, those properties may be slightly dependent on amino-acid sequences.

The infrared (IR) absorbance spectra for 700–7800 cm^−1^ were measured as a function of temperature (26–200 °C) and RH (35–100% RH) by using a FT-IR spectrometer (FT/IR-6100LT, JASCO, Tokyo, Japan) equipped with a Cassegrain microscope (IRT-5000, JASCO). By utilizing a homemade gravimetric system, the change of sample weight was determined for 65–90% RH, and we estimated the average number of water molecules per unit (*N*). The measurement on complex admittance was carried out with a LCR meter (E4980, Agilent, Tokyo, Japan) from 20 Hz to 1 MHz, above that an impedance analyzer (E4991A, Agilent) was employed. From the complex admittance, we obtained the RH dependence of dc conductivity.

## 3. Results

**Figure 2:**
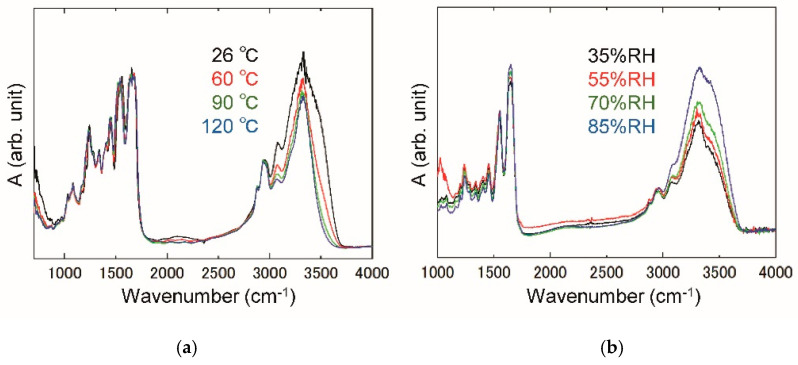
Absorbance spectra at 26–120 °C for 20% relative humidity (RH) (**a**) and at 26 °C for 35–85% RH (**b**).

Figure 2a shows the temperature dependence of IR spectra above 26 °C, at which the RH is fixed to about 20% RH. The integrated absorbance of O–H stretching vibration at 3000–3700 cm^−1^ decreases up to 120 °C, because the water molecule in the primary hydration shell is dehydrated owing to large thermal fluctuations. Below 1500 cm^−1^, several absorption bands originating from the collagen molecule are little dependent on temperature. The absorption bands observed at 1650 and 1540 cm^−1^ are assigned as amides I and II, respectively [29,30]. The former is attributed to a stretching vibration of C = O in a peptide linkage, and the latter an N–H bending mode. With the increasing temperature, the amides I and II slightly shift to higher and lower frequencies, respectively. Those resonance frequencies are slightly dependent on RH at 26 °C, while the absorption band of O–H stretching vibration in Figure 2b exhibits a remarkable variation, which arises from a dehydration.

**Figure 3:**
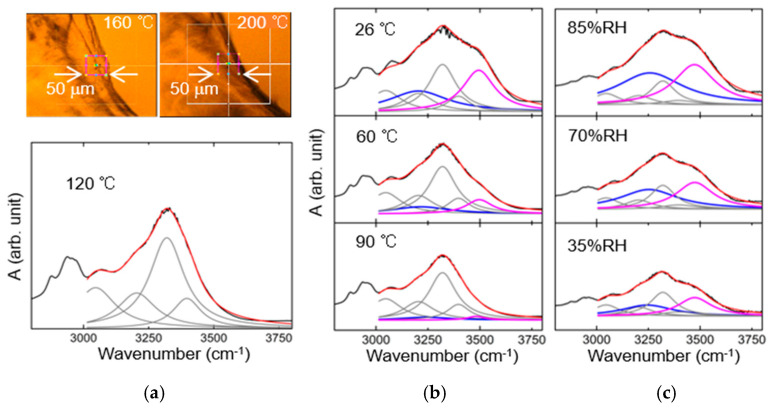
Deconvolution of infrared spectra. In (**a**), the sample shape alters due to a denaturation above 190 °C. The absorbance spectrum at 120 °C is reproduced with four Lorentzian curves (grey curves). Owing to the dehydration of collagen film, the absorption bands due to O–H stretching modes of H_2_O (blue and purple Lorentzian curves) are dependent on temperature (**b**) and RH (**c**).

In order to clarify the hydration state, we have extracted the contribution of O–H stretching vibration in water molecules from Figure 2a,b. At 120–180 °C, the absorbance spectrum is unchanged, and the sample is damaged a little. In the upper pictures of Figure 3a, the sample shape alters above 190 °C because of a denaturation. We have used the spectrum at 120 °C to evaluate the contribution of collagen molecules. The spectrum at 3000–3750 cm^−1^ in the lower figure of Figure 3a is reproduced with a red curve that is the sum of four Lorentzian curves (grey curves). Those components originate from the stretching vibration of C–H, N–H, and O–H groups in the collagen molecule. Those results (grey curves) are used for the deconvolution in Figure 3b,c. As a consequence, we have confirmed the two O–H stretching bands (blue and purple Lorentzian curves), each integrated absorbance of which is almost equivalent, and reveals identical dependence on temperature and RH. The integrated absorbance reflects the number of water molecules hydrated around the collagen molecule. With the use of resonance frequencies of 3200 and 3500 cm^−1^ at 26 °C, the average O–O distances related to a hydrogen bond are estimated as 2.71 and 2.90 Å, respectively [31,32]. Those values are slightly different from the O–O distance in ice (2.75 Å) and free water (2.85 Å) [33,34]. The water molecule corresponding to the 3200-cm^−1^ band is strongly hydrogen bonded to collagen and adjacent water molecules. On the other hand, the 3500-cm^−1^ band implies that the water molecule possessing a nearly-free O–H is connected with extremely weak hydrogen bonds. The band width for those bands is rather broad in all the temperature ranges, and then the O–H stretching vibration may have an anharmonic coupling to the other vibrational modes.

**Figure 4:**
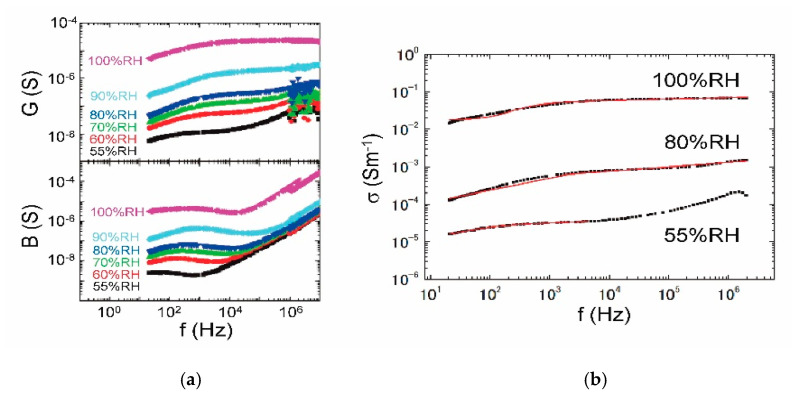
RH dependence of the complex admittance at 26 °C. (**a**) Conductance (*G*) and susceptance (*B*) for 55–100% RH; (**b**) the frequency dependence of ac conductivity fitted with red curves on the basis of Cole-Cole type relaxation.

From the complex-admittance measurement at 26 °C, we obtained the RH dependence of conductance (*G*) and susceptance (*B*) in Figure 4a. The admittance for 100% RH was observed with the sample immersed in water. The conductance below 80% RH is less than 10^−6^ S in all the frequency ranges, and large noise is generated especially above 1 MHz owing to a low accuracy. Taking a Cole-Cole type relaxation into account, the ac conductivity (*σ*) is fitted with red curves above 55% RH in Figure 4b. The *σ* becomes too small to fit below 55% RH. The α relaxation [35,36] caused by a rotational motion of collagen chains is superposed on the proton conduction, which predominates the dc conductivity of a proton (*σ*_dc_).

**Figure 5:**
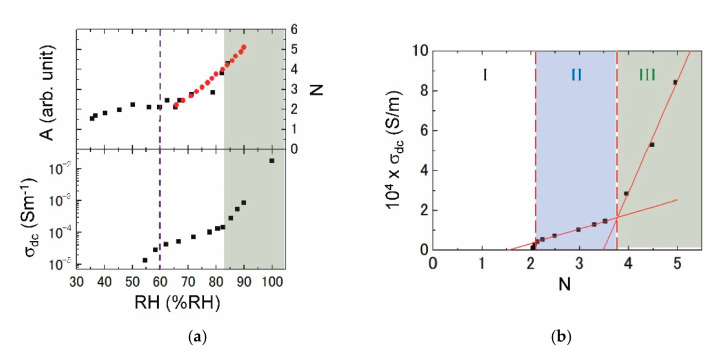
RH dependence of hydration number and dc conductivity (**a**). In (**b**), the dc conductivity vs. hydration number is divided into three regions.

According to the measurement on weight change, we have evaluated the hydration number per unit (*N*). The upper figure of Figure 5a shows the RH dependence of *N* (red circles) incorporated with the sum of integrated absorbance (black squares) due to two different hydration states (blue and purple curves in Figure 3c). The hydration of *N* = 2 changes a little for 50–65% RH, but is slightly reduced below 50% RH. If the *N* is approximated to a linear increase above 90% RH, we may estimate that *N* ~ 7 at least for 100% RH from the extrapolation. The lower figure of Figure 5a shows the RH dependence of *σ*_dc_. The slope changes for 60% RH in agreement with the *N* just mentioned above. Moreover, *σ*_dc_ exhibits a remarkable increase above 83% RH.

From a combination of those figures (Figure 5a), we are available to plot *σ*_dc_ vs. *N* as depicted in Figure 5b. By measuring the three samples in total, we have confirmed the reproducibility of IR spectra and proton conductivity (Figure 5a), and then Figure 5b is approved for the present collagen film. It is concluded that the proton-conducting phenomena are divided into three regions. In region I (0 < *N* < 2), *σ*_dc_ is negligibly small. In region II (2 < *N* < 4), *σ*_dc_ gradually increases with *N*, and thereby a short-range proton transfer is probably permitted. The proton conduction is hugely enhanced in region III (4 < *N*), where a long-range proton transfer may emerge in the whole system. Those three regions are related to the formation of hydration state in the collagen molecule.

## 4. Discussion

**Figure 6:**
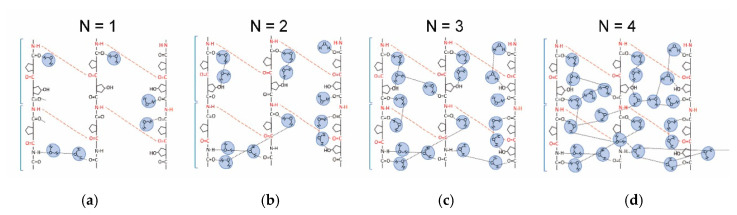
Schematic illustrates of the hydration structure around peptide chains in the form of triple helix. (**a**) Single water molecule per unit (*N* = 1) contacted at the hydration sites; (**b**) isolated intra-water bridge (*N* = 2), in which a proton is basically localized; (**c**) intra-water bridges leading to the short-range proton conduction; (**d**) the helix filled with water molecules, through which proton transfer occurs quasi-one-dimensionally.

We would like to discuss how the water network develops in the collagen molecule on the basis of hydration structures studied previously [22,23,24,25,26,37,38,39,40,41]. The water molecule in the primary hydration shell definitely contacts to the N–H, O–H, and C = O groups through hydrogen bonds as well as in a collagen-like peptide. For *N* = 1, a water molecule is basically isolated as illustrated in Figure 6a, where a triple helix with a repeating sequence of Gly-Pro-Hyp is schematically extended on a plane. Red broken lines represent a hydrogen bond between N–H of Gly and C = O in the different peptide chain. For *N* = 2 (Figure 6b), a proton is fundamentally localized in a water bridge that is partially formed inside the helix. In this paper, a water bridge fundamentally confined in a triple helix is referred to as an intra-water bridge. A proton-transfer pass is hardly built for *N* < 2, and consequently *σ*_dc_ is extremely small in region I.

**Figure 7:**
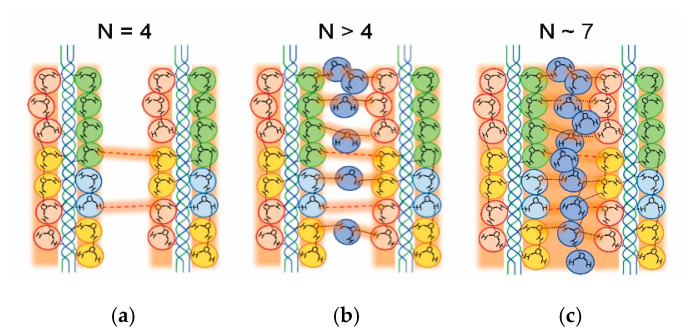
Inter-water bridge formed in between adjacent helices. (**a**) Weak inter-water bridge (red broken lines) because of the large hydrogen-bonding distance at *N* = 4; (**b**) the bridge strengthened with the increasing water molecules at *N* > 4; (**c**) proton conduction via the three-dimensional water network established in between the helices at *N* ~ 7.

At *N* = 3 (Figure 6c), the intra-water bridge is enlarged and connected to each other, and thus a short-range proton transfer starts to develop. At around *N* = 4 (Figure 6d), the helix is filled by the hydrated water molecule, a part of which must spread outside the helix in Figure 7a, where red broken lines indicate an inter-water bridge that directly couples between water molecules hydrated in the different helices. Such a direct inter-water bridge, however, may be weak because of a large hydrogen-bonding distance. In region II, the short-range proton transfer is permitted through the quasi-one-dimensional water networks fundamentally restricted in the single triple helix.

Above *N* = 4 (Figure 7b), water molecules enter into the gap between the helices. The inter-water bridge is strengthened by the gap water molecule. The hydrogen-bonding water network develops three-dimensionally in the film, so that the long-range proton transfer is enhanced with the increasing *N*. As illustrated in Figure 7c, the inter-water bridge is almost established at *N* ~ 7, where the gap is filled by water molecules.

Finally, we discuss the origin of proton as a carrier in the water network. Since a collagen molecule is not an acid, a donor site of proton never exists in the film. In the hydrophilic nanochannel of molecular porous crystal {[Co^III^(H_2_bim)_3_](TMA)·20H_2_O}*_n_*, the water nanotube exhibits a quasi-one-dimensional proton conduction. The neutron-crystal structural analysis makes it clear that the water molecule hydrogen-bonding to the carboxylate-oxygen atom of TMA^3−^ (trimesic acid) tends to dissociate and provides a protonic defect [42]. The self-dissociation generates not only H^+^, but also OH^−^. Those ions are considered to move in an opposite direction with respect to the external electric field. From the remarkable absorption band detected at around 2200 cm^−1^ in the infrared spectrum, the protonic hydrate is assigned as an Eigen-type motif (H_3_O^+^(H_2_O)_3_ and OH^−^(H_2_O)_3_) [43]. The present water molecule in the primary hydration shell contacts to the groups of N–H, O–H, and C = O. On the analogy of the water nanotube, a self-dissociation is possible to occur in the primary-hydration water molecule, and to yield a proton and protonic hole. Further investigations are needed to identify the motif of protonic hydrate.

## 5. Conclusions

Thanks to the systematic investigations of dc conductivity, integrated absorbance of O–H stretching mode, and weight change as a function of RH at room temperature, we have demonstrated that the proton conductivity is strongly affected by the water network formed in the collagen molecule. From the dc conductivity vs. hydration number characterized with the three regions, the proton conduction is negligible in the collagen molecule itself, but dominated by the hydration shell. In region I (0 < *N* < 2), the conductivity is extremely small, and then the proton is basically localized in the primary hydration shell. In region II (2 < *N* < 4), the quasi-one-dimensional proton conduction is generated via the intra-water bridge restricted in the single triple helix. In region III (4 < *N*), the proton-conducting network is extended three-dimensionally by way of the inter-water bridge. For practical use as a fuel-cell electrolyte and bioprotonic device, the collagen film is needed to keep in region III. The filmy samples of sea-bream scale and submucosa [28] mentioned previously exhibit similar proton-conducting behaviors to Figure 4b, and hence we expect that Figure 5b may be a general relation in collagen films.
